# Multiple Sulfatase Deficiency: A Disease Comprising Mucopolysaccharidosis, Sphingolipidosis, and More Caused by a Defect in Posttranslational Modification

**DOI:** 10.3390/ijms21103448

**Published:** 2020-05-13

**Authors:** Lars Schlotawa, Laura A. Adang, Karthikeyan Radhakrishnan, Rebecca C. Ahrens-Nicklas

**Affiliations:** 1 Department of Paediatrics and Adolescent Medicine, University Medical Centre Goettingen, 37075 Goettingen, Germany; 2 Division of Child Neurology, The Children’s Hospital of Philadelphia, Philadelphia, PA 19104, USA; adangl@email.chop.edu; 3 Department of Chemistry, Bielefeld University, Biochemistry I, 33615 Bielefeld, Germany; kradhak@uni-bielefeld.de; 4 Division of Human Genetics and Metabolism, The Children’s Hospital of Philadelphia, Philadelphia, PA 19104, USA

**Keywords:** multiple sulfatase deficiency, formylglycine-generating enzyme, lysosomal storage disorder, posttranslational modification, sulfatases, glycosaminoglycans, sulfatides

## Abstract

Multiple sulfatase deficiency (MSD, MIM #272200) is an ultra-rare disease comprising pathophysiology and clinical features of mucopolysaccharidosis, sphingolipidosis and other sulfatase deficiencies. MSD is caused by impaired posttranslational activation of sulfatases through the formylglycine generating enzyme (FGE) encoded by the sulfatase modifying factor 1 (*SUMF1*) gene, which is mutated in MSD. FGE is a highly conserved, non-redundant ER protein that activates all cellular sulfatases by oxidizing a conserved cysteine in the active site of sulfatases that is necessary for full catalytic activity. *SUMF1* mutations result in unstable, degradation-prone FGE that demonstrates reduced or absent catalytic activity, leading to decreased activity of all sulfatases. As the majority of sulfatases are localized to the lysosome, loss of sulfatase activity induces lysosomal storage of glycosaminoglycans and sulfatides and subsequent cellular pathology. MSD patients combine clinical features of all single sulfatase deficiencies in a systemic disease. Disease severity classifications distinguish cases based on age of onset and disease progression. A genotype- phenotype correlation has been proposed, biomarkers like excreted storage material and residual sulfatase activities do not correlate well with disease severity. The diagnosis of MSD is based on reduced sulfatase activities and detection of mutations in *SUMF1*. No therapy exists for MSD yet. This review summarizes the unique FGE/ sulfatase physiology, pathophysiology and clinical aspects in patients and their care and outlines future perspectives in MSD.

## 1. Introduction

**Figure 1:**
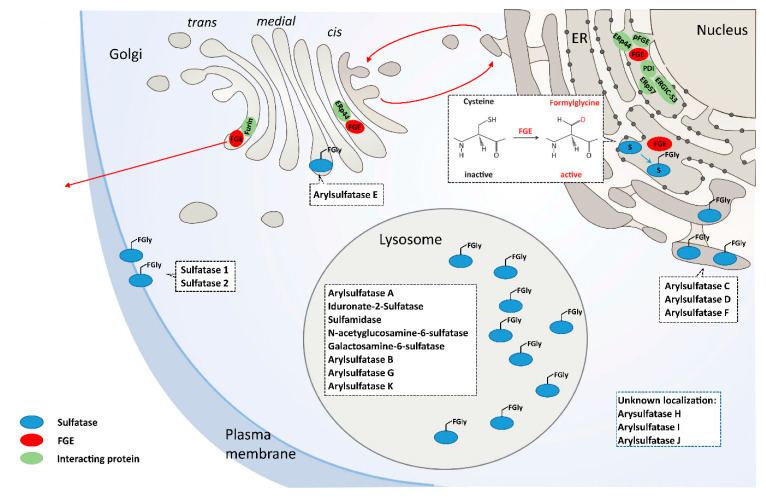
Illustration of the mode of action of formylglycine generating enzyme (FGE). All cellular sulfatases, most of which are localized in the lysosome, need posttranslational activation by FGE in the endoplasmic reticulum (ER) through the conversion of conserved cysteines to formylglycine in their active sites. Few proteins that interact with FGE in the ER and during its secretion upon overexpression in the Golgi are known.

Multiple sulfatase deficiency (MSD; MIM #272200) is an ultra-rare disease caused by defective activation of cellular sulfatases. Despite being the result of a single enzyme deficiency, MSD leads to a failure of a group of enzymes involved in different cellular processes affecting lysosomes and beyond [1,2,3] (Figure 1). Encoded by sulfatase modifying factor 1 gene (*SUMF1*), formylglycine generating enzyme (FGE), the deficient enzyme in MSD, drives a unique posttranslational modification in newly synthesized sulfatases that is necessary for full catalytic activity. 

MSD affects 1 in 1.4 million newborns, and approximately 50 living individuals have been identified [4]. From isolated case reports and publication of small cohorts of individuals, we have an incomplete understanding of the complex phenotype and pathophysiology of MSD.

## 2. From a Variant Form of MLD to a Unique Posttranslational Modification and the Discovery of the *SUMF1* Gene

One of the sulfatases that MSD affects is arylsulfatase A, the defective enzyme in metachromatic leukodystrophy (MLD). Not surprisingly, in 1965, MSD was initially described as a variant form of MLD. In addition to low arylsulfatase A activities, the first described cases also demonstrated reduced activities of different other sulfatases [5]. This was later defined to be a key diagnostic feature of MSD [6,7].

Early attempts to improve the pathophysiology of MSD by genetic complementation of defective or inactive sulfatases were unsuccessful [8,9,10,11]. This led to the hypothesis that a co-factor for sulfatases or a posttranslational modification was missing in MSD. Evidence for a defect of posttranslational modification arose from experiments demonstrating that expressed sulfatases introduced into MSD cells by retroviral transduction were inactive [12]. In 1995, in a breakthrough study, Kurt von Figura and colleagues discovered that sulfatases contain a crucial cysteine in the active site that needs posttranslational oxidation to C-alpha formylglycine for full catalytic activity and that this modification is missing in sulfatases expressed in MSD fibroblasts [13]. The identification of this unique and sulfatase-specific modification initiated the search for the responsible enzyme which resulted in the simultaneous, independent discovery of the formylglycine generating enzyme (FGE) and the sulfatase modifying factor 1 (*SUMF1*) gene in 2003 by two groups using different experimental approaches. The group of Kurt von Figura purified FGE from microsomal fractions of bovine testis. The success of this approach largely relied on the development of a mass spectrometry based FGE activity assay, in which a synthetic peptide, encompassing residues derived from arylsulfatase A active site, served as substrate. FGE activity in elution fractions was monitored for a shift in the mass of the peptide resulting from the conversion of cysteine to formylglycine, until a pure protein could be referred to peptide mass fingerprint analysis identifying bovine FGE leading to its human orthologue. Concurrently, the group of Andrea Ballabio used a microsome-mediated chromosome transfer of human chromosomes into an immortalized MSD fibroblast cell line, narrowing down the genomic location of the gene and identified *SUMF1* through mutation analysis in MSD fibroblasts. Both groups’ results were published in 2003 [1,3].

## 3. FGE the MSD Protein

*SUMF1*, located on chromosome 3p26, is 106 kb long and contains 9 exons, and is ubiquitously expressed with highest levels in kidneys and pancreas. Human FGE is a 40 kDa (374 residues) glycoprotein localized in the endoplasmic reticulum (ER). Residues 1–32 constitute the cleavable ER-signal sequence and the mature protein (residues 33–374) is *N*-glycosylated at asparagine N141. The mature protein is organized into a core domain (residues 73–374) that harbors the active site and an N-terminal extension (residues 33–72). FGE contains 8 conserved cysteines forming 2 disulfide bridges, a pair of catalytically active cysteines in the active site, and a pair of cysteines in the N-terminal extension [14,15]. In cultured cells, FGE is secreted and a major fraction of the secreted form is devoid of this N-terminal extension. The N-terminal extension (residues 33–72) encoded by exon 1 of *SUMF1* is a unique feature found only in FGE from higher eukaryotes and was shown to impart two important functions via its two cysteins C50 and C52. Mutating these cysteines to alanine led to loss of activation of sulfatases and loss of interaction with ERp44 for FGE retrieval back to the ER. For unknown reasons, the N-terminal extension is cleaved off by the *trans*-Golgi-resident furin protease. Other interacting partners ERGIC-53, ERp57 and PDI mediate ER retention, export and retrieval [16]. PDI is of special interest because it facilitates sulfatase activation through gatekeeping of proper FGE folding (see below) [17]. FGE has an interacting paralogue protein pFGE, encoded by the *SUMF2* gene that shares 47% percent of homology. pFGE is co-expressed with FGE in many tissues and binds sulfatases but lacks catalytic function. A proposed function of pFGE is to help FGE activate sulfatase; however, more evidence is needed to support this role [18,19,20]. The intracellular localization and trafficking of FGE along the secretory pathway is considered to be a highly regulated process spatially and temporally controlled by its interacting partners (Figure 1).

The FGE core protein exerts its enzymatic function on newly synthesized sulfatases co- or post-translationally. FGE recognizes sulfatases by a linear sequence motif CxPxR as part of the putative sulfatase signature I (C-STACG-P-STA-R-x(2)-LIVMFW) in the N-terminal catalytic domain. This motif is evolutionary highly conserved and specific for recognition by FGE that converts the cysteine into a sulfatase-specific amino acid, Cα-formylglycine (FGly) [14,21,22,23]. Introducing the CxPxR motif into engineered proteins in cellular expression systems lead to FGly conversion by FGE and is used in a variety of biotechnology applications [24,25].

Sulfatases are a group of enzymes that degrade or remodel sulfate esters. There are 17 sulfatases encoded by the human genome, 13 of which have been characterized biochemically. The majority of sulfatases are localized to the lysosome, while others are located in the ER, Golgi, or on the cell surface (Table 1).

Sulfatase substrates comprise glycosaminoglycans (GAGs), sulfolipids, and steroid hormones [26,27]. In addition, sulfatases exert important regulatory roles on heparan sulfate-dependent cellular signaling pathways [28,29]. High specificity for substrates leads to low redundancy by different sulfatases, as such single sulfatase deficiencies result in severe disorders, most of which are lysosomal disorders [26]. No redundant FGly generating mechanism exists in mammals, and FGE is a limiting factor for sulfatase activation. A complete loss of *SUMF1* or FGE function results in decreased activity of all sulfatases rendering MSD a monogenetic disease [1,3]. The requirement of FGly generation by FGE for catalytic activity of sulfatases has prompted the co-expression of FGE in the production process of recombinant sulfatases for enzyme replacement therapy as well as gene-therapy approaches for single sulfatase deficiencies [30,31,32,33,34].

The role of FGEs secreted form is unknown. A paracrine function has been discussed because of its cellular uptake followed by FGE reaching the ER and exerting sulfatase activation [35,36]. This renders FGE to be one exceptional example of a protein reaching the ER after endocytosis, a mechanism known from toxins or viruses [37]. Further experimental evidence is needed before recombinant FGE could eventually be used for ERT as a therapy for MSD.

Recently, another mechanism of regulation for FGE activity has been proposed: miRNA-95 depletes FGE and results in decreased sulfatase activities leading to impaired lysosomal function, substrate accumulation and a block of autophagy. Lowering the levels of miRNA-95 in MSD patient cells restored sulfatase function exerting a therapeutic potential [38].

The crystal structure of FGE has been established in 2005 and allows insight into FGEs mode of action as well as modelling changes on its structure by MSD causing *SUMF1* mutations [14,39]. FGE has a unique fold with low amount of secondary structure (13% α-helices, 20% β-sheets). Stabilization occurs via disulphide bridges (see above) and two calcium ions. The crystal structure suggested FGE to be a mono-oxygenase as FGly generation consumed equimolar levels of molecular oxygen although no redox active metal ions could be detected [14]. For over a decade, FGE was regarded as a metal-independent mono- oxygenase.

However, an increase in the activity of FGE after in vitro reconstitution with Cu^2+^, led to the proposal that FGE utilizes copper as a cofactor [40,41,42]. Recently, the crystal structures of two prokaryotic FGE-holoenzymes reconstituted with Cu from *Streptomycis coelicolor* and *Thermomonospora curvata* were reported [43,44]. Both structures revealed that a Cu(I) atom is coordinated by two active site cysteine residues in a nearly linear geometry, unusual for copper-dependent oxidases. Based on structural and biochemical data, a structural basis for oxygen activation by the catalytic copper center via a Cu(II) superoxo-intermediate was proposed, thus providing mechanistic insights into FGly generation by FGE and activation of sulfatases.

## 4. SUMF1 Mutations and Functional Consequences

**Figure 2:**
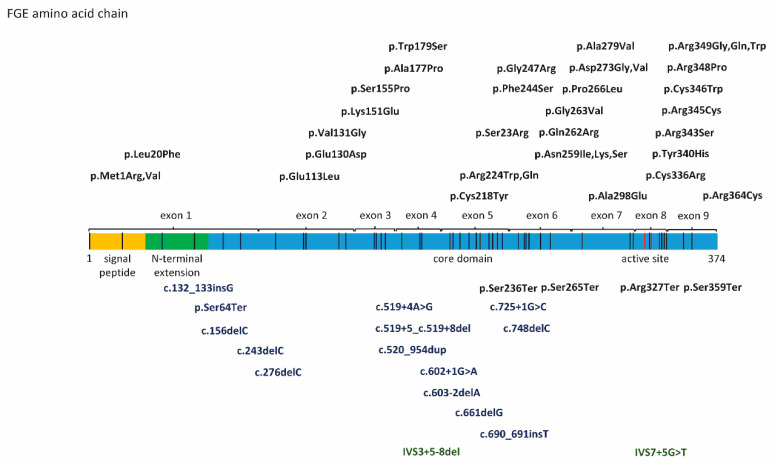
Fifty-three published *SUMF1* mutations. Nonsense mutations (lower panel) and missense mutations displayed as amino acid changes in FGE (upper panel) and their localization on FGE. Exons encoding respective parts of the amino acid chain as well as regions forming the signal peptide, the N-terminal extension and the core domain with the active site are labelled. Mutations are distributed all over the entire length of the FGE amino acid chain without obvious hotspots.

Fifty-three *SUMF1* mutations have been published in the literature since the discovery of the *SUMF1* gene including 21 nonsense mutations (frameshift, stop-gain, deletions), and 32 missense mutations distributed over the entire length of the protein (Figure 2).

Functional consequences of *SUMF1* mutations on the FGE protein have been analyzed for a subset of mutations using a combination of in silico and in vitro biochemical methods. Crystallization of the FGE protein facilitated in silico modelling of amino acid exchanges and prediction of FGE stability and activity especially when variants affect the active site of FGE [14,39]. In vitro experimental data were generated for a subset of mutations using different experimental approaches. Analysis of endogenous FGE levels in patient derived fibroblasts provided information on protein stability and subcellular localization. Pulse-chase experiments of cell lines expressing variant FGE constructs have been used to determine protein half-life for a variety of *SUMF1* variants [45].

In addition to impaired protein stability, FGE enzymatic activity is affected by clinically relevant *SUMF1* mutations. FGE activity is best assessed through the measurement of sulfatase activities at endogenous levels in patient cells or by co- expression of indicative sulfatases and sulfatase activity assays in cell models [45,46,47,48,49,50]. A well-established assay uses bi-directional co-expression of steroid sulfatase and FGE from an inducible vector proven to yield highly reproducible and comparable sulfatase activities referring to FGE activity [51]. Apart from the mass spectrometry-based assay described above, currently there is no assay available for direct FGE activity measurement. Although this approach requires partially purified variant FGE, it has been used for FGE activity determination for a subset of variant FGE proteins [45,50].

The majority of *SUMF1* mutations are of hypomorphic nature resulting in expression of FGE with residual activity [46]. Only nonsense mutations completely abrogate FGE function [52]. All previously analyzed mutations demonstrated reduced intracellular protein levels and excretion supporting the hypothesis that most variants result in early degradation and/or reduced stability of FGE [45,46,47,48,50,52]. In fact, the majority of FGE variants analyzed demonstrate a half-life of less than 2 h [45,49,50,51].

Residual FGE activity of variant proteins was variable ranging from complete loss of activity in active site mutations to 50% of reference activity [45,49,50]. Of note, 1/3 of all published *SUMF1* mutations lack experimental data on stability and activity. Disease causing variants of FGE likely disrupt function through overall instability and decreased enzymatic activity.

Data are beginning to come up on the intracellular fate of variant FGE species. Protein disulfide isomerase (PDI) was shown to interact with wild type FGE and co-expression of PDI improved activation of sulfatases. This led to the suggestion that PDI could play a role in improving proper folding of wildtype FGE [16]. However, PDI was shown to preferentially interact with misfolded variant FGE, which established misfolded variant FGE as a physiological substrate for PDI [17]. Moreover, the crucial function of PDI as a disease modifier in MSD emerged when it was shown to play a role in variant FGE stability and residual activity. In vitro, overexpression of PDI reduces residual sulfatase activities, while PDI silencing rescues sulfatase activities by increasing variant FGE stability.

Downstream effects of FGE deficiency and sulfatase dysfunction are only partially understood in MSD, and only a limited number of studies have explored the pathophysiology of disease. As part of the MSD phenotype, glycosaminoglycans (GAGs) and sulfatides accumulate, as is seen in mucopolysaccharidoses (MPS) and MLD. This abnormal storage results in lysosomal dysfunction, impacting several cellular processes including autophagy [53]. Many pathophysiological aspects proposed for lysosomal dysfunction, e.g., membrane turnover, calcium storage or mTORC1 function, have not been explored in MSD [54]. Effects caused by the remaining uncharacterized sulfatases are yet to be determined.

## 5. Clinical, MRI, and Ultrastructural Features, Disease Classifications, Genotype-Phenotype Correlation

**Figure 3:**
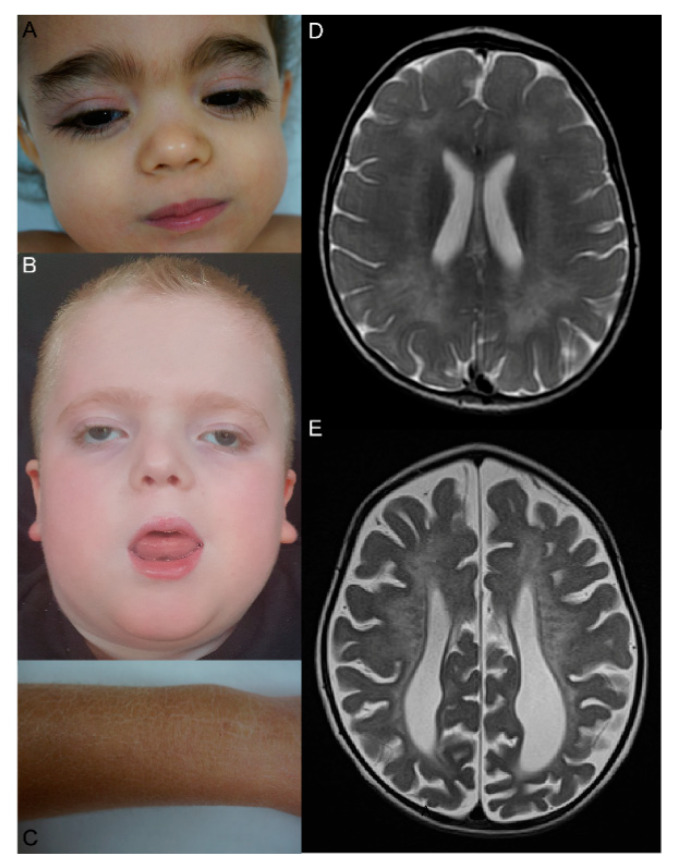
(**A**,**B**): Facial appearance of multiple sulfatase deficiency (MSD) patients. (**C**): Ichthyosis in a MSD patient. (**D**,**E**): T2-weighted cMRI pictures from two different MSD patients displaying typical signs of a leukodystorphy with radiating stripes (**D**) and additional progressive cerebral atrophy (**E**).

MSD is a complex disease because it combines symptoms of single sulfatase deficiencies. It is a progressive, systemic, neurodegenerative disorder of early childhood. The majority of individuals with MSD present with systemic features and psychomotor retardation followed by loss of motor skills, speech, hearing, and vision. Many individuals also have characteristic facial features consistent with MPS disorders, chondrodysplasia punctata, hepatosplenomegaly, and ichthyosis (Figure 3A–C). While all individuals with MSD have neurologic impairment, the other systemic features can be variable and are often not present at birth [45,48,50]. Additional symptoms include cardiac defects, pulmonary involvement, recurrent ear infections, sleeping disorders, hydrocephalus, corneal clouding, retinitis pigmentosa, and hydrops fetalis. Sleep disturbance, feeding difficulties, constipation, spasticity, and hip dislocation are frequent symptoms that affect quality of life. Some patients failed in newborn hearing screening tests. MRI findings can also be variable with demyelination resembling MLD, brain atrophy, corpus callosum hypoplasia, subcerebellar cysts, and hydrocephalus [55] (Figure 3D,E). Ultrastructural findings comprise pleomorphic lysosomal inclusions in Schwann cells detected in skin biopsies, and reduced germ size and hypomineralized enamel in teeth. Brain tissue analysis revealed pleomorphic extracellular, intraneural, and intraglial inclusions and white matter showed gliosis and metachromasia [56,57,58].

Similar to other lysosomal storage disorders, MSD can be divided into neonatal, late infantile and juvenile forms based on age at onset [59]. Revisions of this classification added gradations of disease severity to the age of onset subtypes. While early onset of disease is associated with the most severe forms, it is unknown if how well these subtypes associate with overall clinical outcomes.

Children with early onset disease, the neonatal form, are characterized by presence of multiple MSD-related symptoms at birth. Late infantile cases, which are divided into severe and mild forms, present symptoms in infancy. The latter shows a reduced number of symptoms and a later onset, especially of neurodegeneration and neurological symptoms, and slow acquisition of additional symptoms [45,48,50]. Juvenile MSD is the most rare and the most attenuated form [60].

While these classifications are still in use, there are several limitations to their clinical utility. Upon deeper medical history review, many individuals with an attenuated form of MSD presented with a limited number of symptoms in the neonatal period. These early symptoms often include failure to thrive with feeding difficulties, inguinal hernias, and hypotonia. Based on the existing classification system, these individuals would qualify as neonatal form MSD by onset, but as juvenile or late infantile by clinical course. Clearly, there is a need to define clinically coherent subtypes of MSD that are based on our modern, deeper understanding of the clinical course of MSD [45,48,50].

Sulfatase activities in patient fibroblasts have been used for an alternative, historical disease classification in MSD: Group I fibroblasts exhibit residual activity below 15% of control activity measured in fibroblasts from non-MSD patients. Group II fibroblasts show more than 15% of residual activity, sometimes reaching normal values. Group I fibroblasts derive from severe MSD cases whereas group II fibroblasts originate from attenuated MSD cases [52,56,61,62,63]. Interestingly, and in contrast to group I fibroblasts, group II fibroblasts were reported to show variable sulfatase activity over time in the same cell line. Such cells are likely influenced by cell culture conditions and culture time [61,64]. The mechanisms underlying sulfatase activity variation are unknown. The number of MSD fibroblast cell lines that have been investigated are limited, impairing our full understanding of sulfatase activity fluctuations. However, subsequent publications have supported that a subset of patients could have variable sulfatase activities overtime [48].

Despite the difficulties in establishing a reliable disease classification, the clinical course of MSD falls into two categories: Severe and attenuated. Further work is needed to associate these clinical forms with other features of the disease. There is emerging evidence for a genotype–phenotype correlation in the homozygous form of the disease. Patients with biallelic nonsense mutations have the most severe symptoms and often have absent sulfatase activities (severe or neonatal very severe form) [52]. Attenuated patients often harbor variants that result in a highly unstable variant FGE with low residual activity. Milder attenuated patients can have unstable FGE variants with high residual activity or stable FGE variants with low residual activity. This preliminary genotype–phenotype correlation allows for a rough prediction of the course of disease in cases with homozygous *SUMF1* mutations that have been experimentally assessed [45,48,50]. A genotype–phenotype correlation in compound heterozygous patients has not been fully investigated and is an area of active interest.

## 6. Biomarkers and Diagnosis

Disease specific biomarkers for MSD exploit the biomarkers developed for the related lysosomal disorders. This includes measurements of sulfatase activities, urinary sulfatides, and glycosaminoglycan accumulation. The simultaneous excretion of heparan sulfate, dermatan sulfate, keratan sulfate and chondroitin sulfate is strongly suggestive of MSD. Interestingly, increased GAG excretion is absent in some MSD patients [48] and the factors contributing to such negative GAG measurements in MSD patients are poorly understood.

Sulfatase activities are low in MSD patients, but levels can vary over time, even in the same individual. Also, since sulfatase activities are very dependent on the biochemical assay design, comparing results obtained in different laboratories is difficult [63,65]. In short, a detailed understanding of the association between specific *SUMF1* variants and biochemical phenotypes are lacking. Historically, the clinical diagnosis of MSD was confirmed by measuring simultaneous deficiency of more than one sulfatase activity in patient leukocytes or fibroblasts. Nowadays, *SUMF1* genetic testing supports the diagnosis of MSD. With the advancement of broad based, non-targeted sequencing (next-generation sequencing panels, exome and genome sequencing), it is likely that novel *SUMF1* mutations will be found and the phenotypic spectrum will also evolve.

However, it has to be noted that both biochemical and genetic confirmation have a risk of false negative results. MSD could be missed on panels that measure limited sulfatases, for example, when a test measures only two sulfatases, one could be abnormal and the other normal. In theory, cases could be misdiagnosed as MSD due to arylsulfatase A pseudodeficiency, which is a biochemical artefact of in vitro testing. Sanger sequencing of genomic *SUMF1* could miss large deletions expanding into or spanning the complete *SUMF1* gene. Also, as MSD is so rare, it is possible that pathogenic variants could be mislabeled as variants of unknown significance. Given these diagnostic challenges, we recommend a combination of genetic and biochemical testing with the measurement of at least three sulfatases.

## 7. Care of MSD Patients

No curative therapy is currently available for MSD. MSD patients often face multiple health problems due to the complexity of the disease. As a result from the first International Conference on MSD (Dublin, July 2017) a consensus statement on the complex care and management of MSD patients was published providing the first clinical guidelines for MSD [55]. The consensus statement outlines a standardized framework for comprehensively addressing clinical problems and the management of symptoms in MSD. These guidelines summarize a list of initial evaluations that should be considered in every newly-diagnosed MSD patient to assess for the major clinical complications that have been reported on this rare disease. This includes evaluation of 10 different organ systems, each with a variety of potential complications (Table 2). This comprehensive list of potential interventions needs to be individualized based on the patient and institutional experience. An expert-driven multidisciplinary approach with goal of a high quality-of-life for the affected individuals and also the care-givers should be the primary objective of care [55].

## 8. MSD Animal Disease Models

A *SUMF1* gene-trap knock-out mouse (SUMF1^gt^) was the first MSD animal model. Homozygous SUMF1^gt^ mice displayed absence of eight sulfatase activities tested and storage of glycosaminoglycans in macrophages, kidney and liver. Mice showed signs of head tremor, seizures, skeletal abnormalities and a flat facial appearance. All had congenital growth retardation and more than 90% of SUMF1^gt^ mice died prior to 3 months of age. The mice demonstrated significant systemic and neuroinflammation, indicated by activated microglia, astrocytosis and neuronal loss [66]. Furthermore, fibroblast growth factor signaling was constitutively activated in *SUMF1* knock-out mice derived hematopoietic stem cells and hematopoietic stem progenitor cells resulting in a block in erythroid differentiation, disruption of B lymphocyte differentiation, reduction in mature myeloid cells and aberrant T lymphocyte development [67]. Astrocyte specific knock-out of *SUMF1* in a Cre/Lox MSD mouse model revealed lysosomal storage of substrates and autophagy substrates impairing astrocyte function. This led to neuronal loss thereby highlighting the importance of astrocyte dysfunction in the pathophysiology of MSD [68].

The SUMF1^gt^ mouse model has also been used for the first MSD treatment approach: Combined intraventricular and systemic administration of a recombinant adeno-associated virus type 9 (rAAV9) encoding FGE resulted in a widespread transduction of different tissues followed by an increase of sulfatase activities, clearance of glycosaminoglycans and decrease of inflammation. rAAV9SUMF1 treated mice also showed improved behavioral issues. These results suggest that gene therapy might be a treatment option in MSD [69]. Additional mouse models harboring common human *SUMF1* variants are currently being generated and characterized. These tools can be used to evaluate novel therapies aimed at correcting misfolded FGE species or increasing lysosomal performance and clearance abilities. The high evolutionary conservation of *SUMF1*/FGE has allowed for the establishment of additional animal models. A drosophila melanogaster *SUMF1* knock out fly and *SUMF1* knock out zebrafish lines are currently being characterized. Both models can be used to study early embryonic development in MSD and could also prove useful to future drug screening efforts.

## 9. Patient Organizations and Research Towards a Therapy for MSD

Independently two patient organizations were founded in 2015 by parents with children affected by MSD. MSD Action foundation in Ireland and United MSD Foundation in the US have become drivers in collaborative efforts to improve the clinical care and research options for children with MSD. Since the founding work of these important organizations, two more national patient organizations were founded in Spain and Argentina. Patient organizations have a major impact on basic and clinical research for rare diseases by connecting researchers, clinicians and patients, offering grants for research towards therapy and running patient registries [70]. Social networks connect patients and families with rare diseases globally and facilitate efforts to make rare diseases known to the general public [71,72]. Like other rare diseases, comprehensive natural disease history data on MSD is lacking. Current efforts are underway to better define the clinical course of MSD around the world. Results from these projects will serve as a basis for defining natural disease history and clinical endpoints in anticipation of clinical trials in the near future.

## 10. Future Perspectives

Given the growing interest in the science of MSD and awareness of the disease in general, we anticipate that our understanding of MSD will also continue to develop. The search for additional interacting partners of FGE that regulate enzymatic function, folding, trafficking and degradation will elucidate novel aspects of cell biology. All newly discovered interacting proteins and pathways have the potential of serving as targets for the development of MSD therapy.

In terms of clinical knowledge, definitions of MSD will be informed by ongoing and future natural disease history studies. Such studies may further our understanding of factors that determine disease severity, clarify genotype-phenotype relationships, and reveal biomarkers that can be used to track disease progression. Finally, as newborn screening for single sulfatase disorders and genomic sequencing are more widely utilized, new MSD patients will be identified that will likely expand the MSD phenotype.

The exciting recent progress in MSD research represents the synthesis of basic science and clinical medicine with patient advocacy. As this foundation of knowledge grows, our hope is that potential treatment options will be developed. The growing understanding of the pathophysiology of the disease and generation of the MSD animal models, will set the stage for exploring potential treatment options, design potential therapeutic agents and serve as tools for pre-clinical therapy evaluation and testing. Also, one can expect that meaningful clinical endpoints derived from natural disease history data will aid clinically-relevant trial design. It is crucial that for an ultra-rare and orphan disease like MSD, any therapeutic strategy that ameliorates the disease condition would greatly improve the lives of MSD patients and their families.

## Figures and Tables

**Table 1:** Sulfatases affected in MSD, associated information and disease.

Sulfatase	Alias	Chromosomal Region	Gene	Localization	Substrate	Disease or Syndrome	Abbreviation	MIM No.
Arylsulfatase A	Cerebroside-3-sulfatase	22q13.33	*ARSA*	Lysosome	Cerebroside-3-sulfate	Metachromatic Leukodystrophy	MLD	250,100
Iduronate-2-Sulfatase		Xq28	*IDS*	Lysosome	HS, DS, H	Hunter	MPS II	309,900
Sulfamidase	N-Sulfoglucosamine-sulfohydrolase	17q25.3	*SGSH*	Lysosome	HS, H	Sanfilippo IIIa	MPS IIIa	252,900
N-acetyglucosamine-6-sulfatase		12q14.3	*GNS*	Lysosome	HS, H	Sanfilippo IIId	MPS IIId	252,940
Galactosamine-6-sulfatase		16q24.3	*GALNS*	Lysosome	CS, KS	Morquio A	MPS IVa	253,000
Arylsulfatase B	N-acetylgalactosamine-4-sulfatase	5q14.1	*ARSB*	Lysosome	CS, DS	Maroteaux-Lamy	MPS VI	253,200
Arylsulfatase G	N-sulfoglucosamine-3-sulfatase	17q24.2	*ARSG*	Lysosome	HS	Usher syndrome type 4	USH4	618,144
Arylsulfatase K	Glucuronate-2-sulfatase	5q15	*ARSK*	Lysosome	HS, DS	unknown		
Arylsulfatase C	Steroidsulfatase	Xp22.31	*STS*	ER	Steroid sulfates	X-linked ichthyosis	XLI	308,100
Arylsulfatase D		Xp22.33	*ARSD*	ER		unknown		
Arylsulfatase F		Xp22.33	*ARSF*	ER		unknown		
Arylsulfatase E		Xp22.33	*ARSE*	Golgi		Chondrodysplasia punctata type I	CDPXI	302,950
Sulfatase 1	Sulf1	8q13.2-q.13.3	*SULF1*	Cell surface	HS	unknown		
Sulfatase 2	Sulf2	20q13.12	*SULF2*	Cell surface	HS	unknown		
Arylsulfatase H		Xp22.33	*ARSH*	unknown		unknown		
Arylsulfatase I	Sulf5	5q32	*ARSI*	unknown		unknown		
Arylsulfatase J	Sulf4	4q26	*ARSJ*	unknown		unknown		

HS: Heparansulfate, DS: Dermatansulfate, KS: Keratansulfate, CS: Chondroitinsulfate, H like component.

**Table 2:** Clinical care of MSD patients-systems-based approach.

System	Clinical Concerns
Cardiac and vascular	Arrythmias
	Cardiac hypertrophy
	Cardiac valve issues
	Hypertension
Dermatologic	Hirsutism
	Ichthyosis
Musculoskeletal	Cord compression
	Dysostosis multiplex
	Poor bone health
	Tone abnormalities
Neurologic	Peripheral neuropathy
	Hydrocephalus
	Intracranial pressure
	Seizures
Nutrition and gastroenterologic	Feeding intolerance
	Constipation
	Hepatosplenomegaly
	Gastroesophageal reflux
	Gallbladder issues
Ophthalmic	Cataracts
	Corneal clouding
	Glaucoma
	Retinopathy
	Retinitis pigmentosa
	Optic nerve abnormalities
	Strabismus
Oral	Dental complications
	Hyperplastic gums
	Poor oral- motor coordination
	Tooth enamel abnormalities
Otolaryngologic	Airway obstruction
	Airway narrowing
	Oral and pharyngeal obstruction
	Hearing disorders
	Recurrent otitis media
Respiratory	Obstructive and recessive lung disease
	Sleep issues
	Apnea (central and peripheral)
	Recurrent pneumonia
